# Furred Tongue

**Published:** 1896-02

**Authors:** Edward C. Kirk


ARTICLE IV.

### FURRED TONGUE.

BY EDWARD C. KIRK, D. D. S.

The views expressed by observers with respect to the significance of what is commonly known as "furred tongue" are exceedingly numerous. The color, magnitude, extent, and in fact each varying shade of difference in the appearance of the coating upon the dorsum linguæ has had assigned to it a special pathognomonic relation. The constant association of furred tongue with gastric disturbance is a well-recognized fact; hence it is that when the physician, or in fact the average layman, on examination of the tongue discovers the presence of a furred coating upon it, the diagnosis of a disordered stomach or liver is at once made. There is, however, a wide difference of opinion as to the causal relationship which exists between "furred tongue" and "spoiled stomach;" the popular idea being that the disordered stomach causes the furred tongue. While it cannot by any means be correctly asserted that this is always an error, there is ample reason for calling it in question, and in some instances at least there is good ground for the belief that the relationship is exactly reversed: in other words, that the disordered stomach is produced by and dependent upon the mouth condition.

During the general epidemic of grippe which occurred some years ago, the writer had frequent opportunities for observing the oral conditions of patients who suffered from the disease in question. An almost constant phenomenon which presented itself at the inception of the disorder and persisted throughout its course, was a heavy white coating upon the dorsum of the tongue. The tongue appearance was so constant and characteristic that the observation of this grippal tongue afforded an easy means of diagnosis in the recognition of patients who had been or were about to be attacked by this disease. A somewhat careful study of the phenomena presented by patients in passing through the cycle of the disease compelled the belief that it was zymotic in character, and that the port of entry for the specific organism was generally the oral cavity. In two cases, however, the nasal passages were involved; for an examination revealed a similar white, frosty-looking condition of the mucous membrane lining the nose and naso-pharynx. Immediately following the installation of the specific germ in the oral cavity, local inflammation occurred, involving the tongue, gingival margins, the velum and uvula, pharynx and tonsils. Following this, marked gastric disturbance ensued, characterized by loss of appetite, gastralgia, and the phenomena of gastric catarrh. Later, in some cases,—especially in children,—intestinal irritation of a catarrhal character supervened, characterized by watery passages with, frequently, hemorrhagic symptoms in the shape of bloody stools. These gastro-intestinal phenomena were intercurrent with the general systemic outbreaks, attended with pyrexia and the characteristic nervous phenomena. The cycle of symptoms here outlined plainly indicated the progressive infection of the whole gastro-intestinal tract, from the mouth downward, Here, in these cases, the desirability of thorough mouth sterilization at once suggested itself. To remove the deposit upon the tongue, a number of expedients were resorted to—hydrogen dioxid solutions, diluted Labarraque's solution, sublimate 1-1,000, boracic acid, and tincture of iodin. The application of these remedies was in each case preceded by a thorough mechanical treatment of the tongue by means of a suitable scraping instrument, to remove as much of the deposit in this way as possible. While these efforts were invariably productive of good,—especially noticeable in their effect in preventing relapses of the disorder,—still the tenacious adherence of the deposit rendered its removal extremely difficult, and it was only through the utmost care and vigilance that the growth could be prevented from rapidly reforming, as none of the drugs used seemed to have the power to completely remove the deposit by a single treatment.

Subsequent experimentation in this connection, in trying to remove the furred coating from the tongue in cases ordinarily regarded as due to a bilious or disordered stomach, led to the use of phenol sodique, with most satisfactory results.

By seizing the tip of the tongue between the thumb and fingers armed with a napkin, and drawing it well forward, the furred coating can at once be removed by means of an ordinary tooth-brush, which has been first dipped in water and then had sprinkled upon it six or eight drops of phenol sodique. The strongly alkaline character of this application seems to exert a saponifying action upon the deposit, which is thus easily and completely removed at one treatment.

Thus the modus of infection in the case of grippe is as above outlined, seems to the writer to be beyond question; and further, that many other zymotic diseases—though they may vary considerably in their several local expressions in the oral cavity—invade the general system in an analogous manner. Diphtheria and bronchitis give strong indications of belonging to the class in question, the germs of which have the oral cavity as their port of entry. Hence the extreme importance of systematic and thorough sterilization of the mouth in all such cases; not only as a prophylactic, but as a means of preventing the continuance of the disorder.

The use of phenol sodique, as here indicated, promises most satisfactory results in such cases.—*Dental Cosmos.*

